# Evaluation of the DigiBete App, a Self-Management App for Type 1 Diabetes: Experiences of Young People, Families, and Healthcare Professionals

**DOI:** 10.3390/children10121933

**Published:** 2023-12-16

**Authors:** Nicky Kime, Steve Zwolinsky, Andy Pringle

**Affiliations:** 1 Bradford Institute for Health Research, Temple Bank House, Bradford Royal Infirmary, Bradford BD9 6RJ, UK; nicola.kime@bthft.nhs.uk; 2 West Yorkshire & Harrogate Cancer Alliance, White Rose House, West Parade, Wakefield WF1 1LT, UK; s.zwolinsky@nhs.net; 3 Clinical Exercise and Rehabilitation Research Centre, School of Sport and Exercise Sciences, University of Derby, Derby DE22 1GB, UK

**Keywords:** Type 1 diabetes, young people, APP, intervention, evaluation

## Abstract

Type 1 diabetes (T1DM) is a public health issue for children, young people, and families (CYPF) and requires innovative interventions. The DigiBete app is a self-management and educational app to help CYPF and healthcare professionals (HCPs) manage T1DM, featuring educational advice and resources such as guidance, quizzes, and educational and instructional videos on how to manage T1DM. To assess the impact and implementation of the app, the service-level evaluation deployed a mixed-methods design. App data were captured via the DigiBete platform and an online survey with a non-probability sample of HCPs (N = 178) and CYPF (N = 1165) = 1343. Overall, 55.7% (n = 512/919) of app users were female, and 4855 videos were viewed across the participating areas, with an average of 1213 videos per site (range 776–1679) and 4.4 videos per app user. The most popular videos were how to give a glucagon injection and “My Sick Day Rules”, which showed what to do when CYPF were unwell due to T1DM. Interviews (n = 63) were undertaken with 38 CYPF and 25 HCPs. The findings indicate that CYPF and HCPs found the app an essential tool in the management of T1DM. CYPF and HCPs felt the app provided a valuable educational resource in a central location that was invaluable in an emergency or unknown situation. The app was a trusted and bona-fide source of information that could be accessed at any time. HCPs validated DigiBete in helping CYPF to manage their T1DM. At the same time, the app saved HCPs’ service time and money and helped CYPF take back some of the control in managing their diabetes.

## 1. Introduction

It is estimated that 400,000 people in the UK have type 1 diabetes (T1DM), and this figure is increasing by approximately 4% per year, ranking the UK with one of the highest rates of T1DM in the world [1]. Of this total, 29,000 are children and young people (CYP), representing the highest number of CYP aged 0–14 with T1DM in Europe [2]. Although the literature concerning the risk factors for developing T1DM is well established, the reason why T1DM is increasing is not fully understood, and this is likely to remain the case for the foreseeable future despite ongoing research targeting the prevention of T1DM. Therefore, it makes sense to concentrate efforts on the management of the condition, optimising the care that children, young people and their families (CYPF) receive and ensuring they are supported in the best way possible to improve diabetes outcomes and general health and wellbeing. This includes providing an individualised, integrated package of care delivered by a multidisciplinary paediatric diabetes team alongside self-management [3].

T1DM is one of the most common chronic, lifelong conditions in CYP, and self-management represents a constant challenge, as CYP need to adhere to a daily insulin regimen to maintain appropriate blood glucose levels [4]. People with T1DM spend over 10,000 h per year self-managing their condition, significantly more than the scant 3 h per year interacting with a healthcare professional (HCP) [5]. Given the responsibility for self-management that is placed on CYP with T1DM, it is important that they receive the appropriate diabetes skills and support to be empowered to manage their condition.

One component of the package of support available for CYP is that provided by diabetes technology, namely insulin pumps and continuous glucose monitoring (CGM), including apps that can be downloaded onto a mobile device. In recent years, the number of CYP using technology has increased substantially, helping CYP to better control their diabetes while also reducing the burden of self-management [6,7,8]. Evidence-based standards for diabetes self-management and support advocate the use of technology, particularly engagement platforms such as apps, in what is a developing and expanding market [9]. Indeed, various systematic reviews have shown that apps can help to improve self-efficacy regarding the self-management of T1DM and the maintenance of target HbA1c levels [10,11].

The DigiBete app is a free self-management and educational app and video platform for CYPF and HCPs that was designed as an intervention to help CYPF manage T1DM. The app was designed by DigiBete (https://www.digibete.org, accessed on 13 December 2023) in collaboration with CYPF and HCP. It provides an ever-increasing range of clinically approved and age-appropriate resources to help with self-management, including access to over 200 T1DM films, for example, carbohydrate counting, sports and exercise. In addition, the app offers the ability to store insulin ratios/doses and pump settings and enables the user to receive communications directly to the app from their diabetes team [12]. The app is also available in different languages, including British Sign Language, Arabic, Bengali, Chinese, Polish, Somali, Tamil, and Urdu, and this list has been further extended. The DigiBete app has been introduced in 230 clinics nationally across the UK and is in partnership with the National Children and Young People’s Diabetes Network and the Leeds Teaching Hospitals NHS Trust. It is used by CYP in an age range of 5–16 years and their parents/guardians.

The importance of thoroughly evaluating the implementation of healthcare interventions is widely recognised [13]. A key component of evaluations necessarily includes the perspectives of CYPF and key stakeholders, for example, HCPs [3,14,15]. This is part of a patient-centred approach outlined in the policy guidance provided by the Department of Health [16]. Therefore, this evaluation investigated the implementation of the DigiBete app from the perspective of these key stakeholders, including what worked well and why as well as what aspects of the app did not work as well and the reasons for this. A main driver of the evaluation was to ascertain what needed to be done to refine the app to help support CYPF with their T1DM and to assist HCPs to this end.

Therefore, the aim of this investigation was to evaluate the impact and implementation of the DigiBete app with a focus on assessing the utility of the DigiBete app regarding self-management education and improved outcomes for CYPF with T1DM and to explore its on-going efficacy as a resource for both HCPs and CYPF.

## 2. Materials and Methods

The evaluation was a mixed-method, multi-site service evaluation, and investigative data were collected at sites between 1 November 2020 and 29 February 2023. A partnership approach, where independent evaluators were assisted with some evaluation activities by partners (e.g., DigiBete and the delivery sites), formed the basis of the evaluation framework. This was to gain the views of key stakeholders in the design and delivery of interventions as advocated by Eldredge et al. [14] and the Centre for Disease Control and Prevention (CDC) [13] and, in this case, specifically the experiences of HCPs and CYPF who were using the DigiBete app. The DigiBete app can be downloaded at the following link: https://apps.apple.com/gb/app/digibete/id1488447232, accessed on 13 December 2023). Further information on the app can be found here: https://www.digibete.org/digibete-app/, accessed on 12 December 2023.

The aim of this investigation was to evaluate the impact and implementation of the DigiBete app. To meet the aim, the objectives for the service-level evaluation were as follows:

- To investigate children’s, young people’s, families’, and healthcare professionals’ qualitative experiences of using the app during the first year of care following diagnosis of T1DM in children and young people;
- To identify which parts of the DigiBete app worked well and why as well which parts of the DigiBete app did not work as well and why;
- To collect quantitative data on the use of the app of CYP with T1DM within one year of diagnosis.

### 2.1. Instrumentation and Sampling

Five NHS hospitals across England participated in the evaluation from which participants were recruited, ensuring geographical diversity. At four sites, both qualitative and quantitative data were collected regarding the objectives above. At the fifth site only, qualitative data were available and were collected at the time of the evaluation.

#### 2.1.1. Quantitative Data

Quantitative data for this component of the evaluation came from two main data sources:

1. App data captured via the DigiBete platform (including demographic data, diagnosis history, informational videos viewed, learning undertaken, and awards achieved);
2. A bespoke on-line survey circulated to a non-probability sample of HCPs (N = 178 respondents) and CYPF (N = 1165 respondents) that was designed to evaluate the impact of the DigiBete platform. Participant responses to the survey questions were not mandated; therefore, response rates, and sample sizes are variable for each question.

#### 2.1.2. Qualitative Data

Data for the qualitative component were collected by semi-structured interviews conducted either on the telephone or online via the Microsoft (MS) Teams platform. Semi-structured interviews have regularly been used as a tool to collect information from participants about their experiences of engaging in and delivering diabetes services [3,17,18]. The interview topic schedule was designed based on the main aims of the evaluation and included the same topic areas for all participants. Prior to any data collection, the interview schedule was piloted during the first interviews and reviewed for effectiveness, as is standard practice [19].

Two sample sets were interviewed:

1. Children and young people with T1DM and their families (parents/guardians);
2. Healthcare professionals from the diabetes teams.

A purposeful, non-probability sample of CYPF and HCPs was invited to take part.

### 2.2. Ethics and Recruitment

The required NHS Research and Development approvals for the evaluation were obtained through the relevant Research and Development (R&D) Department at each of the five NHS sites prior to recruitment. Following submission of an application/information, the R&D Departments assessed the investigation as either an audit or a service-level evaluation. Approval numbers were not provided. At their request, evidence of the R&D approvals were shared with the editorial team at *Children*, and this evidence is stored in the non-published materials. CYPF were invited to participate in the evaluation by letter, which was distributed via a gatekeeper through the email function of the DigiBete app. All potential participants were provided with an information sheet and consent form, as per normal practice. Prior to formally engaging in the evaluation, CYPF were required to provide informed consent and were made aware that they could withdraw at any time without giving a reason. They were also informed how they could withdraw from the evaluation.

For HCPs who were members of the diabetes teams, information about the evaluation and what participation involved was promoted to HCPs internally as part of the team’s multi-disciplinary team meetings. Following this, HCPs were invited to contact members of the evaluation team directly to express an interest in taking part in a semi-structured interview. Participation in the evaluation was voluntary. These HCPs were then issued an information sheet, and prior to taking part in the interview, HCPs provided informed consent and were informed how they could withdraw from the evaluation without giving a reason.

### 2.3. Data Analysis

#### 2.3.1. Quantitative Data

Descriptive statistics were used to summarise the socio-demographic profiles of the DigiBete app users and their interactions with the platform. Further, responses from the on-line surveys were summarised to display the characteristics of the responses for each of the samples. Analyses were conducted using SPSS for Windows version 25 (https://www.ibm.com/support/pages/downloading-ibm-spss-statistics-25, accessed on 13 December 2023). These data were only available for collection at four of the delivery sites.

#### 2.3.2. Qualitative Data

A thematic approach based on an interpretative philosophy was used to analyse the interviews and to explore the experiences of CYPF and HCPs using the app, including what worked well and why as well as what did not work as well and the reasons for this. All data from the interviews were primary coded inductively and in relation to a priori themes, namely themes that were identified in advance according to the focus of the evaluation. This is an accepted approach to the analysis of qualitative data investigating participant experiences of health services or interventions [20,21], and it has been used in the analysis of data emerging from the evaluation of diabetes services. Two members of the evaluation team met to refine the specifics of each theme, and initial findings were generated. Throughout the analysis and writing process, the coding, collating, and refining of themes occurred in an iterative way.

## 3. Results

### 3.1. Quantitative Data

Findings from CYPF, using the data sources outlined above, aimed to determine the impact of the DigiBete platform and evaluate the impact of remote support to aid in better diabetes self-management. Results were generated for data that were collected up to February 2023.

### 3.2. The DigiBete Platform

The data were captured via the DigiBete app for the four participating NHS Trust sites and are summarised in Table 1 below. Users of the app were CYP in the age range of 5–16 years as well as their parents and/or their guardians.

Initial findings show that there are N = 1165 CYPF DigiBete app users across the four sites, indicating a wide reach across the service. Data capture for demographic information was variable. However, of the participants providing information, 55.7% (n = 512/919) of app users were female, showing a relatively even split by gender. On average, the app users had been diagnosed for between 3.1 years and 4.2 years, which indicated that the content is valuable in maintaining diabetes control for CYPFs beyond the initial diagnosis period and helpful in sustaining healthy practices. In total, 4855 videos were viewed across the participating areas, with an average of 1213 videos per site (range of 776–1679) and 4.4 videos per app user, which indicated that users were returning to the site to view and digest content.

Regarding the most popular videos, “My Sick Day Rules” (which outlines the actions that CYPF should take when they are feeling unwell because of their T1DM) was the most popular, followed by “How to give a glucagon injection”. There were a wide range of other videos viewed that covered areas from exercise, nutrition, and general diabetes knowledge. This engagement with the videos on the app highlights the cross-cutting appeal of DigiBete, and in turn, this is likely to have reduced the demand created by clinic requests, therefore saving HCPs time and resources. Moreover, these videos are likely to be an integral part of the on-going package of care and continued programmes of education while providing up-to-date information that NICE recommends should be offered to CYPF with T1DM. DigiBete continues to promote access to learning and education to help CYPF manage T1DM.

Not only did CYPFs engage with videos on the app, but they also utilized quizzes and achieved awards, therefore broadening their understanding and consolidating their learning. For example, 902 quizzes were completed across the participating areas, with an average of 226 per site (range of 136–293), just short of one quiz per app user. The average quiz score across the participating sites ranged from 61% to 69%. This learning can aid in developing skills that are essential to living with T1DM and navigating challenges as patients become older.

### 3.3. Survey Results: Health Care Professionals

The following analysis is based on a sample of n = 178 HCPs from multiple sites across the country who provided responses between December 2020 and December 2022.

**Figure 1:**
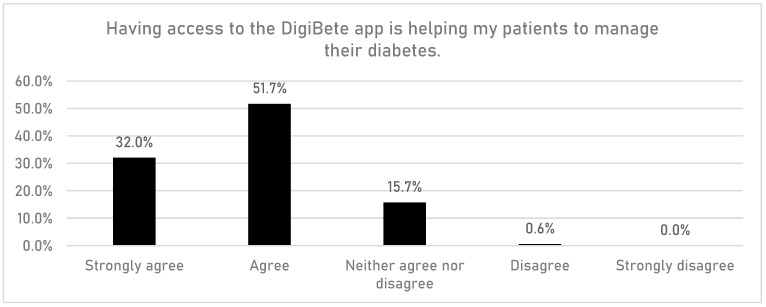
Helping patients manage their diabetes (according to HCPs).

**Figure 2:**
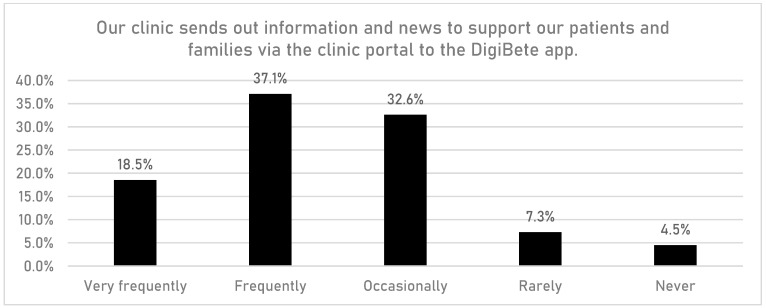
Supporting families via the DigiBete app (according to HCPs).

**Figure 3:**
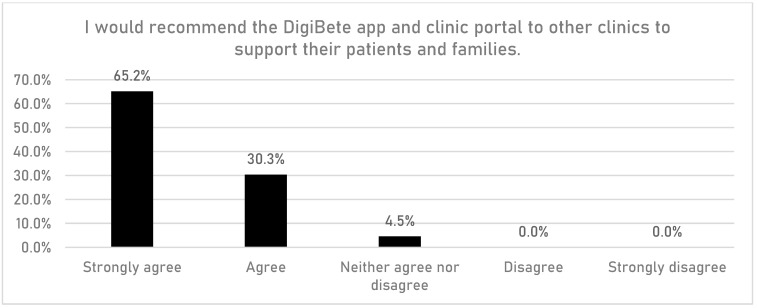
Recommending the DigiBete app (according to HCPs).

**Figure 4:**
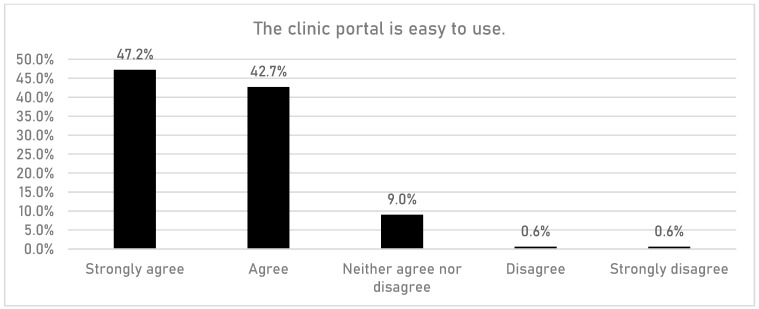
The clinic portal (according to HCPs).

**Figure 5:**
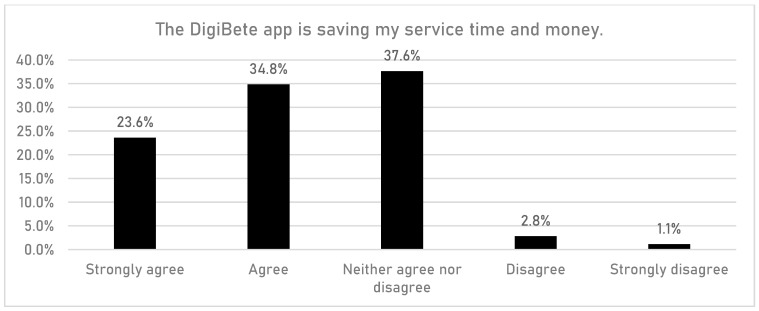
Saving time and money (according to HCPs).

HCPs were asked if having access to the DigiBete app helped patients to manage their T1DM (Figure 1). In total, 83.7% (n = 149/178) of respondents reported that they agreed or strongly agreed with that statement. Furthermore, HCPs were asked if their clinic sent out information and news to support their patients and families via the clinic portal in the app (Figure 2). Overall, more than half of HCPs surveyed (55.6%, n = 99/178) reported that they frequently or very frequently did. The next question asked whether HCPs would recommend DigiBete to other clinics (Figure 3), and 95.5% (n = 170/178) of respondents agreed or strongly agreed that they would recommend DigiBete. Moreover, HCPs were asked if the clinic portal on the DigiBete app was easy to use (Figure 4), and 89.9% (n = 160/178) of respondents agreed or strongly agreed that it was. Finally, 58.4% (n = 104/178) of HCPs agreed or strongly agreed that the DigiBete app was saving their service time and money (Figure 5). In addition, HCPs were asked about the functionality of the app and which parts were most useful in supporting patients. Access to education, quizzes, videos, and resources (30.4%) was deemed to be the most useful, followed by the app creating a place for patients to access their own clinic’s information (20.8%) and having the ability to create groups of patients and send customisable clinic support and updates (20.5%).

### 3.4. Survey Results: Children and Young People and Their Families

The following analysis is based on a sample of n = 1165 CYPFs from multiple sites across the country who provided responses between October 2020 and October 2022.

**Figure 6:**
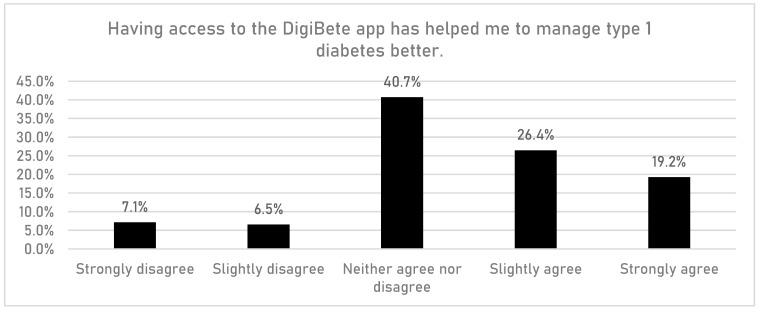
Helping manage diabetes (according to CYPFs).

**Figure 7:**
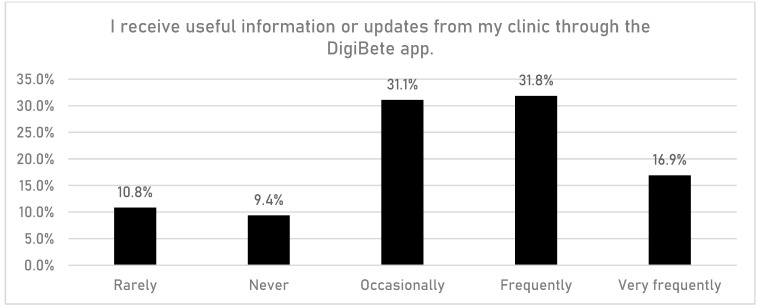
Being supported via the DigiBete app (according to CYPFs).

**Figure 8:**
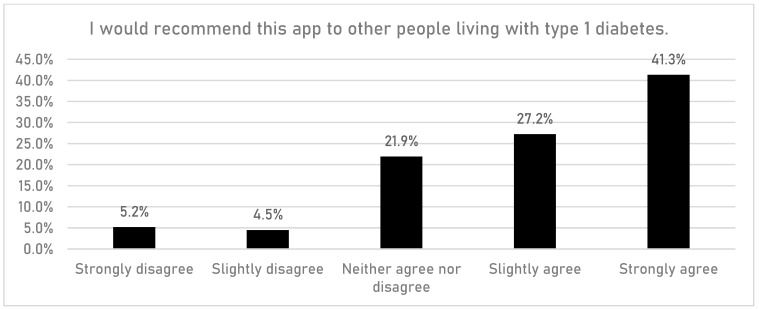
Recommending the DigiBete app (according to CYPFs).

**Figure 9:**
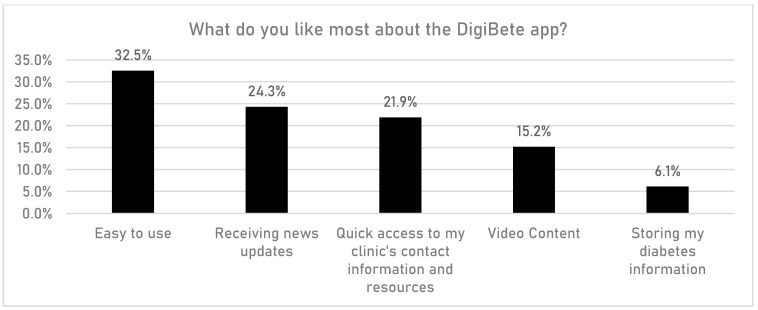
DigiBete app features (according to CYPFs).

**Figure 10:**
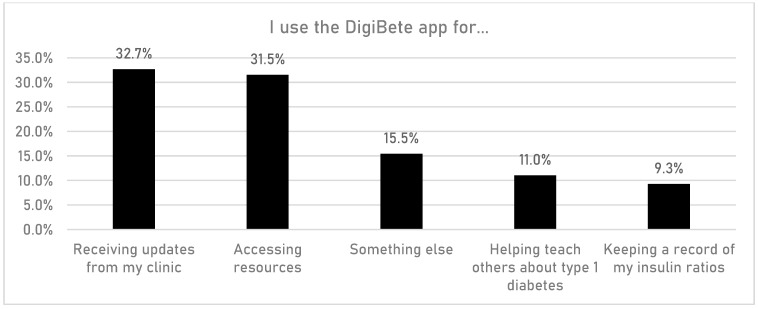
What the DigiBete app is used for (according to CYPFs).

CYPFs were asked if having access to the DigiBete app helped them to manage their T1DM better (Figure 6). In total, 45.7% (n = 532/1165) of respondents reported that they slightly agreed or strongly agreed with that statement. Furthermore, CYPFs were asked if they received useful information or updates from their clinic through the app (Figure 7). Overall, around half of CYPFs surveyed (48.8%, n = 568/1165) reported that they frequently or very frequently did. The next question asked whether CYPFs would recommend DigiBete to other people living with T1DM (Figure 8), and 68.5% (n = 798/1165) of respondents slightly agreed or strongly agreed that they would recommend DigiBete. In addition, CYPFs were asked about what they liked most about the app (Figure 9). The most popular response was that it was easy to use (32.5%), followed by the ability to receive news updates (24.4%) and have quick access to their clinic’s contact information and resources (21.9%). Moreover, CYPFs were asked what they used the app for (Figure 10). Receiving updates from their clinic was the most popular response (32.7%), along with accessing resources (31.5%).

### 3.5. Qualitative Data

In total, n = 63 participants, namely n = 38 CYPF and n = 25 HCPs, were interviewed. The CYP were aged between 5 and 16 years. For HCPs (n = 25), this comprised doctors (n = 3), nurses (n = 14), and other healthcare professionals (n = 8). The results are presented for CYPF and HCPs and grouped according to nine key themes (Table 2) that emerged from the interviews. Selected quotations from the interviews are detailed in this section.

### 3.6. Interviews with CYPF

#### 3.6.1. Acceptability of the App

All CYPF reacted positively when asked about the app:

“I think DigiBete app is fantastic! We are coming up three years since X was diagnosed. It was useful then and continues to be useful now” (Parent).

“I think it’s a brilliant resource and I really wish that we’d had it at the beginning… because I bought a lot of books at the beginning when X was first diagnosed… and those books were overwhelming because you get a whole book. Whereas I think the app is just fantastic that you’ve got all of that in bite-sized, easy to navigate when you’re at the point where you’ve not got the head space to be trawling through things” (Parent).

CYPF liked the visual aspect of the app and commented on how engaging it was:

“The app is really good; it is so not the dreary side of bloody diabetes. When your kid gets diagnosed it is really bloody depressing, it is just insane. I like that it is colourful and positive… it is a nice place to go, not so terrifying” (Parent).

Most CYPF commented on the ease with which they were able to access the app and use the available resources:

“I think it looks really user-friendly… it’s dead easy to find stuff and all the videos that you’d want to watch are there, you don’t have to search very far. And there’s downloadable resources as well… sick day rules, nutrition and your drinking guides, things like that...I’ve found everything that I’ve looked for” (Parent).

However, some CYPF did have issues periodically with opening the app and being repeatedly required to input the clinic code:

“I feel that every time I go into it [the app], I’m having to remember my clinic code. I’m kicked out and then I have to remember to put the clinic code in again. That’s the only thing where I sometimes think, ‘Oh, I can’t believe I’ve got to do this again’, but I don’t know how regularly it resets, but that’s the only thing I would say for me personally. If I could just go straight into the app every time, I would find it a little bit easier to access” (Parent).

#### 3.6.2. Functionality of the App (How Is It Being Used?)

The app was generally the first place that CYPF turned in preference to other apps or search engines. In particular, families of CYP who were newly diagnosed used it as a constant source of reference:

“It was really beneficial, especially when he was first diagnosed. It’s got all of the videos on. We got loads of like hints and tips from the nutrition side of things, little snacks that he could have. When he was switching from his insulin pen to the pump, there was videos about that. So, the videos were really, really helpful at the beginning… I got loads of information from it” (Parent).

Families of CYP who were not recently diagnosed used the app more as a way of keeping up to date with the latest news and information. They tended to look at the app a couple of times a week, on a weekend, or before going to bed. Also, families used the app as a reminder of what to do in specific situations such as when their child was sick or having a hypoglycaemic attack:

“I looked at the one where they’re having a hypo and needing an injection. I refresh that every now and again and I’ll re-watch that one because I’ve never had to use it and I just want to make sure that if I ever did, I know how” (Parent).

In addition, CYPF used the app as a way of finding out answers to questions or specific information straightaway rather than contacting a member of their diabetes team, whom they said they were sometimes reluctant to contact in case they were busy with the demands of their work within the services they provided. CYPF stated that they did not necessarily want to bother their diabetes team with their concerns, and therefore, the app provided an alternative means of reassurance:

“I think it’s a really good system. It’s like that little thing on your shoulder going, ‘You can do this. If you’re not sure, go and check it out’. It’s that little piece of security rather than having to try and get hold of somebody on the phone or emailing and waiting for a response. So, for the new people coming through now, it’s gonna make their lives a lot easier, for newly diagnosed people as well as old hands like me. I think for newly diagnosed people, it’s that safety net they need” (Parent).

CYPF liked to have control over their diabetes management and did not always see the need to contact their diabetes team when they could find out the information themselves from the app:

“I suppose now, just like refreshing my memory. I wouldn’t want to bother the nurses… we tend to just manage ourselves a lot more now” (Parent).

Most CYPF found it easy to navigate the app. They spoke about the app being the way forward given the emphasis on mobile technology:

“For kids they are so techy… so for them to lead up to when they are adults and manage their own long-term condition, I just think it [DigiBete app] is the way forward cos they’re just so brilliant at it. You’re speaking their language really, aren’t you?” (Parent).

#### 3.6.3. App Content

##### Videos

All the CYPF used the app to watch the videos. CYPF commented on the accessibility of the videos, and they liked them because they were concise:

“They’re not long videos. They don’t take a lot of time to sit and watch so I tend to do that while I’m sitting in bed of an evening and have a quick check out and see what’s new” (CYP).

They appreciated the specific videos that were aimed at different age groups, particularly for older children who were becoming more independent and for whom the videos on alcohol, emotional wellbeing, learning to drive, and sexual relationships were especially relevant. Parents stated that they would sit down with their son/daughter at an appropriate time to see what advice was available for young people. Also, most CYPF watched the videos that were more applicable in a time of crisis:

“We’ve looked at quite a few of them. I’ve used them to show my husband about how to load up the glucagon pen and the sick day rules ones, the hypo and hyper” (Parent).

##### Quizzes

CYPF thought the quizzes were more appropriate for younger children, and as was the case with the videos, they needed updating regularly, especially if they were to be used as an ongoing educational resource to learn about T1DM:

“He likes the quizzes. These could be done with being updated, because they are the same; there is a need for selection of quizzes so we can see progress” (Parent).

##### “My Sick Day Rules”

Many CYPF turned to the app for the “My Sick Day Rules” in preference to anything else. In an emergency, one parent commented on how the app was easy to follow with its clear, up-to-date information:

“Having that [“My Sick Day Rules”] at the press of a button was fantastic. It was easy to follow, and I was scared about following it cos it’s something we’ve never done before, but it worked brilliantly for us and yeah, I think prevented us from having to get in touch with HCPs who were busy at the time, and it kept X well” (Parent).

##### Age-Appropriate Information

Another feature of the app was information divided into age categories. CYPF found this especially useful, and it helped them to find their way around the app more easily. In addition, they thought it was useful to have photos of the doctors and nurses on the app so they could see who their HCPs were, which made it more personal, particularly for younger children:

“The main thing I liked about it straightaway was how it was split into age categories, so you could go to like stuff that was appropriate for your child… and the fact that it’s quite personal, people on there that he knows… I was able to show him the videos and he recognised the doctor that was on there” (Parent).

CYPF were aware of the videos with recipes and stated that because the recipes were on the DigiBete app as opposed to another app, they felt confident trying them and would be more likely to vary their diet and cook new recipes.

In terms of content, CYPF liked the fact that when they logged onto the app, the most important information was there immediately:

“What I do like about it is, I’ve never had to do an emergency injection on X and that’s something that I’m hoping I’ll never ever have to do, but I’m glad when I log onto that [the app], it’s the first visual I can see. If I was in a panic at least I can go straight onto that” (Parent).

#### 3.6.4. Behaviour Change

Many CYPF believed the app had had an impact on their diabetes management due to the ease with which they were able to access information:

“Before it was a case of like let’s just get on with it, but now you can go on there and there’ll be an answer somewhere in the DigiBete videos… because of the information that’s on there now, most of the answers to the questions you’ll have are on there, which is really useful to have in one place” (Parent).

Also, the app helped to increase the confidence of CYPF and their ability to self-manage because they knew it was an approved and credible resource:

“I know it’s there, I know all the information on it is right, relevant, so I guess it’s just a trustworthy source rather than just Googling anything” (CYP).

“Honestly, I know that they [DigiBete] know what they’re talking about. You know, it’s what they do. It’s correct” (Parent).

“I really think it’s a brilliant app. It’s a great resource to have… I’m sure it would affect a lot of people if it didn’t exist because it does have a positive effect on the management. No, it’s really good, brilliant” (Parent).

Young people who were beginning to take more control of managing their diabetes spoke about having the app on their phone and being able to see the DigiBete icon. It acted as a reminder while also helping to normalise their diabetes management:

“Without it [DigiBete] being an alarm that gets annoying… it’s a quick jog of the memory without feeling that it’s a nag sort of thing… I think with it being a reminder, I’ve got better at managing my diabetes cos I know I’ve got better at doing my insulin before I eat a meal rather than during or after. I know it’s helped me doing that” (CYP).

Parents believed that the app would also further motivate young people to manage their diabetes:

“Because she has that information, she’s more empowered to try and take some ownership with her diabetes. She tends to have a little bit more control of her ratios and she looks at it [the app] more… so she’s taking more ownership for her own diabetes really” (Parent).

Furthermore, they thought the app helped to normalise T1DM by providing information on aspects of a child’s or young person’s life beyond the medical management of the condition:

“The app normalises things for them [CYP] so I think it’s wonderful… I think it covers those things that are really important to the child specifically like, how can I manage going to a sleep over? To a child that’s so crucially important, but might not be something that clinically would be” (Parent).

The “My T1D” area of the app was helpful for storing important information and as a back-up in case a child’s/young person’s pump failed. CYPF used the app to keep a record of HbA1c levels and to monitor these over a period of time:

“In particular I use it to log X’s HbA1c numbers so I can keep track of where X is going… logging their HbA1c so you can do something about it, if they’re going up or if they’re going down” (Parent).

Others commented that they experienced difficulties inputting information correctly into the “My T1D” area:

“When I’ve tried to fill some things in, to upload, it sometimes doesn’t allow you to save it” (CYP).

One of the overriding themes was the reassurance for CYPF provided by the app. Many CYPF commented on how much they relied on the app and the comfort it gave them just knowing it was there:

“I do think people would struggle without it to be honest. Just like for the reassurance. Like I would have been on the phone to X a hell of a lot if I didn’t have it [the app]” (Parent).

“They [diabetes team] don’t work every day so I think I’d panic if I didn’t have it [the app] … I think it’s like your bible really, isn’t it? Like I say, I don’t use it daily, but when I need it, it’s there” (Parent).

“When you go onto Google or anything like that, you can Google all these things about how to treat, but you don’t know whether it’s from a reputable source, but at least I know when I go on there [the app] it’s something that my team have recognised as important and they know the content of it so they know the content is appropriate. So, it feels like a safe area to get information rather than typing something in Google and hoping for the best” (CYP).

#### 3.6.5. Benefits of DigiBete

In addition to the specific attributes outlined above, the other benefits of the app were the updates and notifications that the CYPF received, for example, the information about the diabetes staff who were on call on a weekend and the ability to schedule clinic appointments within the app. CYPF found this information really helpful as well as timely reminders throughout the year, for example, prompts alerting them to the clocks changing. For many CYPF, the benefits of the app in helping young people to manage their diabetes in the future as they became more independent was an important concern:

“I think it’s really handy and useful and may be as she gets older, it’ll be more practical for her rather than me needing it, cos she will have to take over it [the app] herself at some point. It’ll end up being her comfort blanket as well” (Parent).

“I think for X, she’s probably started using it more in the last kind of year onwards now that she’s starting to become more independent with it all. So, I’m pleased that she’s got access to that information without necessarily having to go through me or through the team. She can start accessing more information about different subjects on there. I would be concerned that if she didn’t have kind of an app that was regulated, that she could be getting incorrect information from the internet and that this is like a safe place for her to go, specifically aimed at children as well for the right age group” (Parent).

A couple of parents worked in education and suggested how valuable the app was in terms of training staff:

“Because the videos are so specific you can look up exactly what you need to look up and just watch that 5 min video. You’re not having to trawl through a great big training programme. Some of our online courses and videos last forever, but these are great, because they’re really quick, just whatever you want to know videos, aren’t they?” (Parent).

“I’m just thinking about the DigiBete website and how useful it would be for staff [in school] to see some time, cos on the app I’ve seen the dictionary with all the terms in. That’s really useful. It’s got a lot of videos that are helpful for doing training with staff” (Parent).

Likewise, parents advised teachers in school to look at the app:

“I’ve actually used it with a couple of the teachers at X’s school. Before she’s gone away on school trips, I’ve said, ‘Look, go on DigiBete, go onto this section and it will tell you exactly what’s what’. And I’ve found that the resources on the app for those type of people… I was like, ‘Oh yeah this is great’” (Parent).

#### 3.6.6. Addressing Inequalities

Most CYPF accessed the app using their mobile phone and personal data allowance or home broadband package. They tended to look at the resources on the app in real time rather than download them to look at another time. During the COVID-19 pandemic, the app was particularly helpful. For example, the videos were freely available so CYPF could watch them at any time in their own home:

“If you get stuck and you’re on your own, you’ve got the videos to watch and to understand diabetes. Right at the beginning it’s a minefield, just to learn as much as you can until further down the line and then you start to understand it more, but they’re still there just in case cos you know what happens with diabetes, things change” (Parent).

For some families with newly diagnosed CYP, they were unable to go to a clinic during the [COVID-19] pandemic. Also, CYPF commented that in lockdown, it could be harder to contact their diabetes teams. In both instances, the app was invaluable:

“I suppose it contributed to helping us in the beginning and helping us manage it when obviously it, the hospital wasn’t as accessible as it should have been, with it being lockdown. Although X was on the other end of the phone, it did help us quite a lot” (Parent).

“I used it a lot for the My Sick Day Rules. Obviously, it was hard sometimes to get in touch with the diabetic team cos obviously working from home, etc. so I used it. It’s my ‘go to’ for the My Sick Day Rules and it’s my ‘go to’ for the emergency injection” (Parent).

#### 3.6.7. Potential App Improvements

Overall, CYPF were extremely happy with the app, and many were unable to suggest any ways in which the app could be improved. However, there were areas where some CYPF thought the app could be tweaked.

1. In relation to newly diagnosed CYP, families proposed the following:

“So, I think it was just, when you first get diagnosed, you get bombarded with so much information… There’s quite a lot of information on that front page and quite a lot of ink… I think when I first opened it I just closed it again, cos I thought, ‘I just can’t, I don’t know where to start with it’… So that was like my initial response. When you’re just in that mode of getting so much information, you almost want somebody to take you by the hand and go, ‘Right, this is the important bit to go to first and then the rest of it you can look at later’” (Parent);

“If it had on the initial page a section just for newly diagnosed and this is what you should look at first… sick day rules, treating hypos, how to do the injections, things like that, I could have worked my way through a more methodical way” (Parent).

2. For CYPF who were not recently diagnosed, they wanted more detailed information and direct links to additional information:

“I do think there could be a bit more in-depth information on there. There’s quite a few times where I’ve seen something and I’ve thought, ‘That’s interesting, I’ll read that’, and then it just scratches the surface and really provides information that I already know rather than the more detailed” (Parent);

“And I know sometimes it refers to, you can get more information at Diabetes UK, but then there’s not a link and to be honest, I’m quite a proactive person, but I’m still lazy. So if there was a direct link, touch this link and it will take you to the exact place you want to go, then I’d be more likely to do it rather than go onto another website and have to search for it… Yeah, it mentions that there are these links, but you want it there and then rather than have to go off and try and search for it” (Parent).

3. CYPF had some further suggestions for making the app more user-friendly:

“I did think there’s an area where you can add in the HbA1c. I think it would be quite useful to add in a height and weight record as well just because I write it down in my notes on my phone at the moment and I need the most up-to-date weight to go into her pump so it is useful to have it and to keep an eye on her progress as it were. Also, I saw there was a bit where you could add an appointment, but I put it straight into my phone. Unless you could have the ability where you put the appointment into the app and then it automatically updates it to your calendar, I probably wouldn’t use that because it’s just another thing to do. Where it said, ‘time in range,’ it would be nice to add a date for that. At the moment you just put one figure and you can’t add multiple entries and it would be useful to like track it” (Parent).

4. CYPF wanted to see some changes to the content of the videos:

“It [the video] was just saying, ‘Some foods have high GI, some foods don’t,’ ‘They’ll have an effect on your blood sugars’. And then it didn’t say anymore. Well, I know that… Rather than just touching on it, it would be really useful to have then a link to the foods with the GIs and what that actually means and how you would actually deal with that in real terms with insulin dosing. We get it all from the hospital, the dietitians, but it would be really useful to have it on the app” (Parent);

“I did the quiz which I thought was quite good, but if you don’t get the answers right, it doesn’t tell you what the correct answers are… It says, ‘Go back and watch these videos’, but the videos that it suggested didn’t answer the questions I got wrong. One of the videos didn’t work or wouldn’t work for me last night. So, I just think if you got a question wrong it would be useful to tell you the answer straightaway and then a bit of an explanation as to why that was the answer” (CYP).

5. CYPF suggested that to encourage more young people to watch the videos, they needed to be a lot shorter, as CYP did not have the required attention span to sit down and watch lengthy videos:

“She’s speaking TikTok language; she’s so easily swayed just by spending time flicking through videos… Children now, they’re not bothered about watching a 20 min, half hour programme. It’s all very like instant, short, like messages, videos, music, something to look at. So, I wonder whether or not there’s something in that, you know if the videos were much more snappy she might be more like encouraged to have a flick through them” (Parent).

6. CYPF thought some of the content needed revising to make it more age-appropriate, for example, resources for children and young people in the younger and older age ranges:

“Not that many videos for under 5′s and the quizzes and things aren’t available for under 5′s… now it’s quite limited in what we can do with it” (Parent);

“Content for young adults probably needs to be a bit more… quizzes are a bit babyish, not really aimed at young adults. They don’t really want to do a quiz so I think that needs may be a bit of thought, how they want the young adults to view the information that’s necessary for them” (Parent);

“When you transition to adult clinic, I can’t see the adult team. I think that would be useful for me, you know to get to see familiar faces so that when I walk in, I know who everyone is” (CYP).

7. Several CYPF thought there needed to be more information on the app relating to mental health and wellbeing:

“A lot of the young people, if they think they’re just gonna go on and see a lot of people, you know happy, smiley, which is true, that does happen. But you know, I feel there is an area to develop if you’re finding it really tough and adding that as a resource and putting the links on there to the mental health services” (Parent).

8. CYPF suggested a search function for key issues such as carb counting, alcohol, etc.

9. CYPF were happy that they received notifications about new articles or updates but suggested that the timing of notifications could be tweaked:

“I don’t know if it’s possible, but to ping it [the notification] outside of school hours so that when it comes up on their [CYP] phones, they can access it straightaway rather than them forgetting about it” (Parent).

10. Some CYPF had queries about where they could locate information in the app:

“In our hospital section it says the team and it goes through all the members of our hospital team, but you can’t see the full job description… when you click on there, it still doesn’t give you their full job description at the top” (Parent);

“There’s a big tab at the bottom that says, ‘My Clinic’ and I thought that’s where the news would be to say who’s on that weekend, but the ‘My Clinic’ tab has just got the general directions that are in all the time about just where the clinics are and the team… So I suppose if it’s news just from your clinic it would make sense for it to be in ‘My Clinic’ section as well. It could be in both areas, couldn’t it? Notifications and it could come up in ‘My Clinic’ area” (Parent).

11. CYPF queried whether additional functions could be added to the app:

“It would be quite useful if the app spoke to other ones to save you having to repeat the information on several different apps” (Parent);

“I know I’m four years in, but I’m still not confident to just go and change basal rates and things like that… I don’t know if it’s something that could be put on there [the app]… a little bit of guidance… I feel like I need a comfort blanket all the time. If there was something that could be put on there” (Parent).

12. Some CYPF thought that CYP would benefit from being able to interact with others on the app:

“I wonder if there’s a way of the kids being able to interact with each other. Especially at the moment with lockdown there’s a lot of the social aspects taken away from the condition that they’ve all got… for the kids to be able to communicate” (Parent).

### 3.7. Interviews with HCPs

#### 3.7.1. Acceptability of the App

In general, HCPs were positive about the app:

“We have come such a long way in such a short space of time. Three years ago, there was nothing and this [the app] is incredible” (HCP).

HCPs reported that it was mostly the newly diagnosed CYPF who were using the app:

“A lot of our new patients are using it because we’re introducing it to diagnosis, but those patients who’ve been diagnosed quite a long time, I would say are not really using the app at all” (HCP).

HCPs stated that CYPF generally found the app easy to use. This was especially the case for those who were regularly engaging with the app:

“I think for those people [who are using the app] they find it easy to use and if you’re regularly using it, you’re probably know where everything is, don’t you? I think it’s maybe for the people who don’t use it that often, that they maybe think they don’t really know how to navigate it” (HCP).

HCPs thought that some CYPF did not realise the benefits until they started using the app:

“I have one Mum who has been up against T1D for years… and she is anti-technology. It is you know, a middle-age thing, like me, can’t do technology and she says, ‘I just can’t do it, can’t do it.’ She finds it easy to manage her child’s diabetes now that she’s warmed to the technology to help her. She is using it [the app] for notifications and everything else, so I think the app is straightforward” (HCP).

HCPs reported their use of the app coincided with the changing times in relation to technological advances:

“The fact that it [DigiBete] is an app. Time is changing and becoming more technological, everyone has a phone and so apps are accessible. Giving out bits of paper is old fashioned; no one reads them. They can access an app in their own time and all the videos and information, videos and tips are there, and everyone has access to a phone” (HCP).

In some cases, HCPs felt that they needed to familiarise themselves with the app but felt supported by DigiBete:

“The app was a bit tricky to begin with, knowing and being able to generate the posts and attach the things. Not quite knowing the way that it works out. However, whenever there’s anything that we’ve struggled with, whenever you feedback to them, they [DigiBete] really listen to the feedback, so they’ve changed the things that we’ve said we find tricky” (HCP).

HCPs thought that the app was very helpful at diagnosis, when there was a lot of information being shared with CYPF. Information that was in one place and in a consumable format was most helpful to CYPF:

“It’s [the app] just really become more and more like our one place where we direct everybody to it for everything. It’s also been useful in terms of helping us stay with the families, contacting them when we have an on-call system, so all our teams’ details and things are there” (HCP).

“I think having all the information in one place is great and I think having those videos which can then go alongside what you’re saying” (HCP).

#### 3.7.2. Functionality of the App (How Is It Being Used?)

As reported previously, HCPs felt the app was mostly used by newly diagnosed families:

“We use it at kind of first diagnosis. We are trying much harder now and we’re doing it more well, more and more at every clinic… We’re finding that our new families are really taking it on board, cos it’s just helpful, isn’t it? Whereas our old families who’ve lived with diabetes for a long time, it’s harder to kind of engage them into it because they don’t know it and they don’t see it. I don’t think until you use it you see the benefits. Well, I think it’s so fabulous” (HCP).

HCPs stated that the app was used to help facilitate learning in the clinic, as a talking point, and to signpost CYPF to relevant information:

“With our structured education for our first six weeks of our newly diagnosed patients, I not only say get them to look at the essential’s videos, but I also get them to have a look at kind of our clinic sick day rules as well. Because even though the children aren’t sick yet, it’s winter and they will become sick at some point soon” (HCP),

HCPs said they encouraged CYPF to record information on the app, but in some cases, this was an exercise that they needed to continue to work on:

“We’re also trying our best to use ‘My TID’ for them [CYPF] to record as much data as they possibly can within clinic. That’s not going quite as well as we hoped, but we will keep plugging it” (HCP).

HCPs reported that they used and signposted CYPF and others to the essential videos:

“It’s good for the age of the children using the videos to try and engage the parents with the topic of education when they came to clinic” (HCP).

“There are quite a lot of schools that are looking at the videos using the school’s part of it, not just the essentials, but you know, the brilliant little films around the different key stages and listening to people who have been there and done it” (HCP).

All HCPs stated that they used the app to mail information and keep CYPF updated on news and events that were happening locally and that could be of help to CYPF:

“We started using it [the app] initially because we could automatically contact a big group of people. It lets us send correspondence and things to the families. Printing and stuffing envelopes takes a lot of time and costs a lot of money” (HCP).

“We have used the app to post information, such as how they [CYPF] can access wellbeing services. We have posted praise after young people got their GCSE exam results and updated people on staff being on annual leave. In the nursing team, we all post. If we become aware of an event happening locally that is beneficial or supportive to the families, we post that. Mainly we use it for posting and we use it with newly diagnosed families and tell them to get this app on their phone. It is brilliant” (HCP).

#### 3.7.3. App Content

HCPs reported that the essential videos as well as the HbA1c tracker were helpful to CYPF:

“They have all the essential films which is great and having all the other films that are on there as well, so the food and drink and particularly the technology” (HCP).

“They all really like the Hb1c tracker in there [the app], where they can see the progress or hopefully the good progress of their levels” HCP).

HCPs also reported the benefit of recording the doses and pump settings in the app:

“In the past we were writing all the numbers down in little books which parents then loved… And now we can put this, the doses, and the pump settings or whatever on ‘My Type 1’ part of the app. I think when they know where that is, that’s you know, all of those options are really straightforward for them” (HCP).

HCPs felt that the app helped CYPF in an emergency or unfamiliar situation:

“The app is helpful because it’s, you feel like it’s an extra level of security that they’ve got” (HCP).

HCPs also reported on the care plans:

“The care plans have been really helpful. People who are using it [the app], I think they’ve been early to kind of get them completed and get them [CYPF] set up” (HCP).

HCPs were keen to emphasise that while the app was seen as helpful in conjunction with face-to-face support from the HCP, the app did not provide all the information for CYPF about their T1DM and could never replace the personal interaction between CYPF and HCPs:

“It’s helpful in conjunction with real face-to-face… It’s okay as a 24h contact, but you still need someone to talk to and make the context right… sometimes it needs to be talked through to ensure family understands what it means… It’s not a standalone piece of tech; it’s not going to replace us completely” (HCP).

#### 3.7.4. Behaviour Change

HCPs reported that CYPF who were newly diagnosed and engaged with the app from the beginning were most likely to change their behaviour. However, those who were less “tech savvy” were more difficult to engage:

“We’re almost preaching to the converted. The people that really are engaged with their diabetes are using it [the app]. The ones that aren’t particularly tech savvy still won’t download it, or if they download it, it’s not used at all. So, it’s difficult to keep plugging it to the people that aren’t [engaged]” (HCP).

In addition, some HCPs stated that they had to be selective about initiating behaviour change with some CYPF or teenagers, who could be more resistant to engaging with the app:

“Some of them [CYPF] are pushy and say, ‘Show me the DigiBete app, what is it then?’ and they get to it [the app] quite quickly and others… you have to pick your arguments really in clinic, especially with some of the teenagers” (HCP).

In some cases, HCPs reported the ongoing challenge of promoting the app and feeling exacerbated that some CYPF did not adopt a resource that they felt was very helpful to them in managing their T1DM:

“We are a bit bemused as to why people don’t use it [the app]. I can understand the teenagers. I think our frustration in our clinics is why do people not use it more? It’s quite curious. I think if I was a parent of a child who had diabetes, I would find it helpful cos you can put so many things in there. They should be able to seize it. Maybe they don’t realise… we just need to keep saying, ‘This is what we use, and this is it’” (HCP).

HCP also reported that engaging newly diagnosed CYPF was not as difficult:

“We’re lucky in our site, we’ve had a great uptake [of the app]. I think we were 100%” (HCP).

An inability to log into the app was an issue for CYPF, and HCPs thought this was often used as an excuse for not using the app. Therefore, HCPs used time in clinic to re-engage CYPF with the app as a way of managing their T1DM:

“The more we can drip-feed them [CYPF], the more likely they are to use the app. If we show them, they may go back into things such as exercise or alcohol and the implications for diabetes. The more we use it as an education model, then that is the best way to get families to use it” (HCP).

“When they’re logged out, they just don’t do the steps to log themselves back in again. But when you ask them, ‘Do you like it [the app]?’ ‘Oh yeah, yeah, I really like the app,’ so it’s just like having to drip feed them all the time with logging in” (HCP).

#### 3.7.5. DigiBete Champions

Some HCPs were formally recognised as DigiBete champions by their colleagues, and they encouraged fellow HCPs to promote the app to CYPF:

“I have a team of nurses who are very into this sort of thing [promoting the app] and they do most of the education for our patients. They sort of grabbed the DigiBete app as a great opportunity when it was launched and when we were allowed free access to it, they started getting all of our new patients onto DigiBete. And at the point of diagnosis making them download the app in hospital” (HCP).

HCPs reported being systematic in their approach to promoting the uptake of the app to CYPF:

“We are quite a small service, one of the smaller services in the country. So, we’ve been quite systematic that we can plan to see every patient about something, and we systematically approach them all about the app. We didn’t let them [CYPF] leave the department until they’d downloaded the app or had them all set up” (HCP).

HCPs reported that the DigiBete champions took actions to help their team promote DigiBete in their work:

“The PSDN [Paediatric Specialist Diabetes Nurse] X has been very proactive about using the app and has really sort of embraced it. So, she’s printed out sort of laminated sheets that we can have on our desks in the clinic room… That’s got the QR code that they can scan to get the app and they’ve got the clinic code and just seeing that as a visual reminder” (HCP).

“I am trying to guide the patients into different sections such as the videos. The more we can guide people, the more we can get the CYPF to use the app. I am trying to show the other nurses how to do this and I think this is the best way to get the families to use the app. The more we can show the families the content, the more they become aware. One parent said, ‘If you didn’t show that, I would never have looked at this’” (HCP).

#### 3.7.6. Benefits of DigiBete

When HCPs were asked about the benefits of using the app for CYPF, they commented on the wealth of readily accessible information:

“Its ease of access, tailored, specific for the age, with the benefit of immediate communication for us to them and a place to store settings for quite complex technology. So, that is what I think it’s best for really, tailored self-help” (HCP).

HCPs stated that the app had helped CYPF deal with a particular situation or an unknown event:

“The ‘My Sick Day Rules’ has all the information. The fact that this is there probably helps with some young people. We don’t have loads of admissions, but the emergency phone number is in the DigiBete part of the app and that and ‘My Sick Day Rules’ has helped families” (HCP).

“If they [young people] are in a situation. Maybe they’ve been out drinking with their friends or they’re at a house party or something and they don’t want to phone Mum for help and wake them up, it’s [the app] there. So DigiBete is a bit of a safety net or a get out of jail card!” (HCP).

HCPs reported that the app was a trusted source of credible information for CYPF, and they used it rather than unauthenticated sources of information:

“I mean we’re lucky, we have a 24 h on call so they can get hold of us, but I think before now we hear stories of people going onto, you know the Facebook parent forums and getting info from other families who have been there, done it. And that is a little bit worrying sometimes. But when you know that they’re first thinking of the DigiBete app…” (HCP).

“So, when they are first diagnosed, they really like it, I think. So, when we show it to them in hospital, they’re completely sort of like, ‘Wow, this is great!’ and I think it is. It’s a reassurance to them knowing that there is something there with them. They don’t have to keep picking up the phone to us” (HCP).

An important advantage of the app reported by HCPs was that the app could help teenagers manage their T1DM independently:

“There are kids up until 12–13, their parents tend to be the people owning the diabetes and possibly looking for the resources on DigiBete, whereas I like to think it’s a good place to go to for the teenagers if they have a question. If they know they’re going to go out with their friends getting drunk. You know having somewhere where they can go and look at it without having to have the shame of asking an adult how to cope with drinking. It might be their get out of jail card for young people at midnight one night when they’re trying to understand what to do as they grow in independence” (HCP).

#### 3.7.7. Addressing Inequalities

Most of the HCPs we spoke with reported that access to mobile phones to use the app was not a significant barrier even in the families that were less well-off:

“Most people have got a phone. I haven’t come across anybody who doesn’t have a phone, even though we’re talking about poverty proofing and all kinds of things nowadays” (HCP).

“Now all of our families have got smart phones and then they’ve got Wi-Fi access at home, so they might not have credit, but they have Wi-Fi and cause it’s an app you can put it on a tablet as well, can’t you? So, I don’t think there’s many of our families that have not got a phone. The only thing is, like when a lot of them do swap their phones a lot for new contracts. Yeah, but I don’t think that’s an issue because even though they’ve not got credit, they’ve all got Wi-Fi” (HCP).

While, there was an acknowledgement that some CYPF faced inequalities, diabetes teams took measures to begin addressing access issues and increasing awareness:

“Yeah, I think that it is potentially an issue for some families in X. We’re using some of our tech money that’s coming to level up regarding pumps and CGMs [continuous glucose monitors], to provide mobile phones. So, when we give them [CYPF] a phone to access this CGM, it will also have DigiBete on. It will upload those essential apps beforehand. Whether they again use them and also looking at those families who are in sort of quantile 5 and can’t afford to buy SIM cards. It’s how we can support those through charity monies to obtain SIM cards” (HCP).

“We are trying to make sure with the poverty proofing that the families who are less well-off have access to a mobile phone with the DigiBete app on it. We are trying to make sure if their phone is incompatible, we can address this” (HCP).

HCPs reported that sometimes, they served families for whom English was not their first language:

“I mean, in our area we don’t have many families who don’t have English as their first language. It’s quite a sort of white English population, but I know for lots of other areas it isn’t. You know, for one or two of our families, it’s been fantastic being able to sign persons at the app and being able to have all those resources in a different language. So yeah, I think it’s a fantastic positive” (HCP).

HCPs provided examples of CYPF who experienced difficulties and inequalities, including the following:

“One of my families who’d had diabetes for a while, they were a family who struggle with learning, with diabetes and with life in general. I did a home visit, and the young person was unwell and used the DigiBete app. Mum acted straight away and used the app. Before the child would have been hospitalised and she was absolutely delighted that she’d managed the situation at home with the My Sick Day Rules from the DigiBete app” (HCP).

HCPs highlighted the ‘My T1D’ section of the app as being very helpful, especially during the pandemic and at times when CYPF could not necessarily visit the diabetes unit in person. The app was seen by some HCPs as an extension of the service they provided:

“I think it is great that we can put our newsletters and things on there [the app] and give information out particularly during COVID-19 when everyone was worried about that and how it would affect them… for getting information to people” (HCP).

“We were sending out a lot of updates, particularly when stuff came out nationally about national [COVID 19] guidance and when they heard that diabetes made you more at risk… We were having to send information out to reassure people… It has saved us a lot of time and money as well” (HCP).

“We just simply wanted to secure their self-management education resource. We wanted them to have their insulin ratios stored in a place where they could access it and just feel that they had the clinic in their pocket if you like, when accessing clinic [during the pandemic] could be trickier for them” (HCP).

#### 3.7.8. Potential App Improvements

In general, HCPs were extremely positive about the app, but they did offer suggestions to improve the app.

1. There was a consensus amongst HCPs about CYPF feeling frustrated that the app logged them out:

“The only thing I think that is not difficult but that is a little downside, is the logging into it [the app] all the time. Whenever there’s an update on your phone, or I’m thinking an update by DigiBete in some way, then you’re locked out. I can get myself logged back in quickly, but sometimes families struggle with that” (HCP).

2. More training needs to be provided to increase CYPFs’ awareness that a “forgotten clinic code” button is available. DigiBete has added this function to improve the experience of CYPF. This requires CYPF to input their DigiBete account email address, after which they will instantly receive a code to prompt a password reset.

3. Some HCPs reported that they would like to have the ability to log in as a professional:

“I think it would be useful to have a way of logging in [to the app] as a professional and having access to all the information about all the different age group things that are on there” (HCP).

4. HCPs reported that they would find it useful to have a search button:

“It would be useful to have a search button. Because unless you know your way around the app, you don’t know where things are. And I think, especially for some of our new ones, if they want to look up something then they find it a little bit tricky” (HCP);

“When I was trying to get someone to look at like the section about carb counting where they’ve got those takeaway menus, I literally could not find the search button on there to get to show them that, so I had to then go to the website to show them and I still don’t know to this moment in time where that is actually, which is bad isn’t it?” (HCP).

5. Another area where HCPs thought there was room for improvement was the notifications:

“How you get notifications. It says they’ve posted a new article or something. So, on my app it says I’ve got 18 notifications. Now, where can I find those 18 notifications? You’ve gone to the notification bit, and it says, ‘There are no notifications’” (HCP).

6. In some instances, HCPs reported that the videos were too long for teenagers and perhaps needed to be presented in a different format:

“I’ve got two teenagers myself, so TikTok are 30 s, so maybe some sort of teenage TikTok diabetes, I don’t know. But yeah, they generally won’t sit and watch a 5min video, but they will watch 30 s and flick on and flick on and flick on. So maybe that’s something, but that’s me making that up. Not from the teenagers” (HCP).

7. Another suggestion for improvement was using the app for appointments, which some sites are already doing:

“Because I know I keep saying to people, that appointments were going to be fed through the app at some point and that was going to be linked up. I don’t know if that’s still going to be happening. It would be quite helpful because I think that sometimes it’s quite a hook… Sometimes when people are sort of not signing up and you’re really kind of keen that they’ve got that information to say you know, ‘Our appointments will soon be coming through this system [the app]’” (HCP).

#### 3.7.9. Resource Savings Associated with DigiBete

When asked if they felt the app has helped save financial resources, HCPs reported the following:

“Yes, we keep a track of it [the resources] and we kept a spreadsheet with how long it would take us to send like a mail shot to all our patients. How many bits appear around how much printing, envelopes posted that kind of thing, but also how long it would take to print and stuff and label envelopes up. So, we’ve tracked that for a long time. How much nursing admin time and how much money it’s saved. It’s thousands. I have a small case load and it has been thousands” (HCP).

“I mean, I think it’s a massive cost saving. I think that’s the way to try and sell it [DigiBete]. That it’s a huge cost saving because it’s saves human beings having to be paid to reiterate the same message again and again and again. If we’ve got a simple ‘go to’ resource, it makes us use our time much more efficiently” (HCP).

“DigiBete is extending our reach, saving us time and hopefully we’ll improve the uptake of those care processes and hopefully that will be a quality improvement that we can make over time that will have a legacy into adulthood” (HCP).

## 4. Discussion

Diabetes technology for use by young people and their families can optimise T1DM management and outcomes from an early age [22]. Disease management requires interdisciplinary care coordination between CYPF and HCPs as well as others involved in the care of CYP with T1DM [22]. The importance of thoroughly evaluating the implementation of healthcare interventions is widely recognised [13]. To the best of our knowledge, this is the first service-level evaluation of an app for the self-management of T1DM to investigate the perspectives of CYPF and HCPs in the use of DigiBete, a digital app for self-management of T1DM. The key findings indicate that CYPF and HCPs found the app an essential tool in the management of T1DM. CYPF and HCPs felt the app provided a valuable educational resource in a central place, and it was invaluable in an emergency or unknown situation. Furthermore, the app was a trusted and bona-fide source of information that could be accessed at any time, and it helped CYPF take back some of the control in managing their diabetes. In a climate of scarce health resources, HCPs felt the app had been a contributory factor in saving the NHS time and money. For example, almost two-thirds of HCPs agreed or strongly agreed that the DigiBete app was saving their service time and money. In addition, CYPF and HCPs identified areas for improvements, which shared with the developers so they can refine the app.

The NHS has been profoundly affected by the availability of mobile devices and apps in recent years, especially since the onset of the COVID-19 pandemic. More than 90,000 new apps were added to app stores in 2020 [1]. The rise in popularity of apps has enabled patients to engage with the health system through virtual visits, track their general health metrics, and, importantly, monitor and manage their health condition or symptoms remotely [1]. Resource issues and capacity have been some of the major drivers of this shift as well as the need for better communication and fast access to information at the point of care. The DigiBete app provides a “one-stop-shop” intervention to help CYPF and HCPs.

### 4.1. How the DigiBete App Was Used by CYPF and HCPs

The NDPA Spotlight Report highlights that, in general, CYPF are benefitting from using diabetes-related technologies to manage their condition depending on the paediatric diabetes service they attend [6]. Indeed, in this study, CYPF reported that the app helped them change their behaviour to manage their T1DM. Furthermore, during the COVID-19 pandemic, CYPF and HCPs reported using the app to access information on lifestyle and to check “My Sick Day Rules” at a time when there were restrictions on what people were permitted to do and where people could go. This extended the reach of the diabetes services. Alaslawi reported that the potential for technology to become an integral part of T1DM routine care has been accelerated due to the pandemic, a situation which resulted in reduced or non-existent face-to-face CYPF and HCPs interactions. Indeed, CYPF reported that having the app during the pandemic was very reassuring, especially for parents who were worried about their child having a diabetes episode and needing to be able to access trusted information about what to do in unknown circumstances [23].

HCPs reported that the app was helpful in their practice when working with them in clinics and at home, particularly for newly diagnosed CYP. Some HCPs found that they needed to be both flexible and creative when encouraging CYPF to use the app. With some CYPF, HCPs were constantly having to promote the app and had to consider the readiness and preparedness of CYPF to engage in the app. HCPs sometimes needed to adopt negotiating strategies for encouraging CYPF who might be more resistant to using the app. HCPs referred to “picking and choosing their battles” with more reluctant, resistant, or rebellious young people in terms of when would be the right time, if at all, to encourage their use of the app. Even if CYP were not prepared to engage at that point, the app provided the facility for them to re-engage with the content when they were ready to do so at a later stage.

### 4.2. The Importance of the DigiBete App

For the CYPF, the app was helpful in navigating the sheer volume of information that is available on T1DM, especially when first diagnosed, which can be an overwhelming and stressful time for CYPF. The app provided CYPF with an opportunity to go back and retrieve information on managing their T1DM effectively and at a time when they were in the necessary “headspace” and could more readily process the information they required. As the information was available online, it was centrally available to all users and seen as a determinant to better self-care, which was appreciated by CYPF. Furthermore, they could access answers to their questions immediately rather than having to call HCPs, and they used the app frequently to refresh their knowledge on relevant topics, for example, how to give an injection. CYPF reported that the app helped them take back some control and autonomy when managing their T1DM, especially when recently diagnosed or when they were away from their normal routine, for example, on a family holiday or school trip. CYPF also said the app was helpful for people who were looking after their child, specifically when a child was on a school activity during which a teacher needed more information or when a grandparent was looking after their grandchild. Families of younger children used the age-specific videos, and HCPs encouraged parents to watch the videos with their children to help facilitate learning and development. For teenagers, research has identified that adolescence is a time when young people seek to achieve increasing independence and to separate emotionally from their parents, prioritising relationships with their peers [22]. The app was seen as a tool to help young people make the transition from being dependent on their parents to achieving independence and managing their own T1DM.

Our findings indicate that CYPF found the app reassuring in this respect, knowing that CYP could access key information on the app whenever they needed it, for example, managing T1DM after consuming alcohol. An additional advantage of the app was that it gave CYP autonomy and meant they did not have to speak with an adult or one of their parents if they chose not to. Moreover, HCPs informed us that some CYP were reluctant to engage with them in the clinic, and therefore, the app provided an opportunity for CYP to access the information they needed when they were ready. CYPF found the videos on recipes helpful for young people when they started university and needed to cook for themselves. However, one of the most important ways that CYPF used the app was for the “My Sick Day Rules” to help them manage an unknown situation or when they were feeling poorly due to their diabetes. HCPs felt that having information in an app that was easily accessible meant that some CYPF were more likely to use the app as a first port of call for information to self-manage their T1DM rather than ringing the clinic straightaway. HCPs reported that there were CYPF who, in an unknown situation or T1DM crisis, had used the “My Sick Day Rules” in the app initially instead of contacting their diabetes nurse or clinic, This not only helped CYPF manage their situation, but it also helped save scarce NHS resources such as HCPs’ time. However, CYPF knew to contact their diabetes team if they had additional concerns and/or the problem could not be addressed by using the app. It should also be stated that the “My Sick Day Rules” feature in the app aims to supplement NHS guidance, which recommends CYPF contact their diabetes team if they have a “hypo”. Furthermore, HCPs also reported that the app was a trusted and bona-fide source of information that CYPF could consult for correct advice. This contrasted with web-based resources, which could not be relied upon to be accurate, potentially leading to the worsening condition of CYP and more urgent medical intervention.

HCPs felt the app was an important tool to be used alongside regular clinic visits, during which they could direct CYPF to the many different features of the app, for example, videos, “My Sick Day Rules”, and key HCP contacts. However, some HCPs reported that they were required to constantly nudge CYPF to use the app. This was important in helping CYPF to appreciate the benefits of the app, which became increasingly apparent the more the CYPF used it. Furthermore, in the context of limited healthcare resources, the app meant HCPs could send out group notifications when previously they would have had to “stuff and label” envelopes to send to CYPF. Interviews with CYPF and HCP indicated that learning had taken place, and this was reflected in examples of CYPF who amended their lifestyles as well as the practices that enhanced their preparedness for managing their or their child’s diabetes. It would be valuable to add to the learning identified in this evaluation in future research and to further investigate both the depth of learning that took place and how this was best facilitated.

### 4.3. How the DigiBete App Could Be Improved

Overall, CYPF and HPCs were very positive about the app, but as with any intervention, our service-level evaluation identified areas for improvement. This reflects the benefits of undertaking an evaluation of an intervention from the perspective of key stakeholders [13,14,15] and a genuine aspiration to improve the app and better serve the needs of CYPF.

Lupton [24] reported that CYP appreciate the availability of information online and the opportunities to learn more about their bodies and how to improve their health and physical fitness. They enjoy being able to connect with peers and they find emotional support and relief from distress by using social media platforms, for example, YouTube and online forums. Research has indicated that CYPF are active users of digital health technologies, but it is notable that they still rely on older technologies such as websites and search engines to find information [24]. Therefore, it is important in an evaluation of the DigiBete app to examine how CYPF use various platforms for accessing information on different content. CYPF identified an area for improvement in the app, which was the ability to communicate with other CYP who had T1DM. They stated that such a feature would have been particularly advantageous during the pandemic when lockdown restrictions were in place. However, this poses additional considerations regarding monitoring and safeguarding in the use of the app and the associated resources involved in these activities.

As well as increased interaction with CYP with T1DM through the app, CYPF reported that it would be beneficial if the app could facilitate real-time engagement with HCPs. This was seen as especially important during the pandemic when some CYPF experienced increased isolation [25]. Clearly, interaction with HCPs through the app poses an additional resource challenge for services that are already working to full capacity [26].

Furthermore, CYPF suggested they would benefit from a print function on the app to enable them to print the newsletters and important information; a link to the quizzes in order to see if CYP were learning from the content on the app; the ability for the app and a diabetes pump to “speak” to one another to avoid input error; and the ability to share their T1DM care plan with other people, particularly staff in schools. Also, CYPF wanted to see links to mental health support and content focused on the management of T1DM in relation to key life events, for example, starting school, going to university, and starting a new job.

HCPs suggested that the usefulness of the app could be increased by considering the order in which content appeared on the app, particularly for those who were newly diagnosed; syncing clinic appointments through the app; and incorporating more images to accommodate the needs of those individuals for whom English was not their first language. Helping to make the app an integral part of diabetes services has been identified as important for ensuring clinical effectiveness. However, while some apps support HCP engagement/consultation, many others have little or no involvement with the healthcare team [8,10], Our results demonstrate that this was not the case for the HCPs and the DigiBete app. The HCPs that we interviewed did engage with the app; indeed, HCPs or DigiBete champions were often the main drivers and promoters of the app, especially with newly diagnosed CYPF. For example, HCPs used the app on a regular basis to communicate with CYPF and post notifications for their attention. In this respect, the DigiBete app differs from most other apps, which is one of its unique selling points, as emphasised by both the CYPF and HCPs.

Evaluating public health interventions is important to help improve their delivery [27]. Having highlighted the different improvements that CYPF would like to see, our findings have identified that CYPF have different needs and expectations of the app, which can pose a challenge in catering to everyone. On-going communication from HCPs to CYPF, in terms of what to expect from the app, becomes even more important when promoting its use [28]. Therefore, in facilitating engagement, working with HCPs to make apps useful for the routine care of CYP is a good investment. The involvement of end-users in research can enhance its quality, relevance, credibility, and legitimacy [27,29]. Brew-Sam advocated an integrative approach involving CYP, parents, and health care providers to develop future technology [22]. Feedback from these groups is regarded as extremely valuable when improving health systems to meet the needs of CYPF. However, this can result in rising expectations of CYPF regarding their T1DM care. Our findings and the literature demonstrate how important it is that CYPFs’ expectations of the app do not translate into additional demands on HCPs, many of whom already report increasing workloads [26].

### 4.4. Inequalities and Use of the App

When delivering technological interventions, an important aspect to consider is that of inequalities in technology. This includes the ability of CYPF to afford smartphones and the functionality to access apps and a home Wi-Fi connection. These issues were not widely apparent in our evaluation, and similarly, the absence of ability to access IT hardware and software were not evident in the CYPF we interviewed. However, HCPs reported that they experienced difficulties when accessing the app on ageing departmental phones and/or slow organisational Wi-Fi connections. These are important considerations when developing technology-based interventions [30,31]. Furthermore, we asked HCPs if CYPF experienced difficulties with buying a smartphone and purchasing data for accessing the app. In most cases, this was not an issue. HCPs reported that having a smartphone was regarded as a basic requirement if not a necessity. Some NHS Trusts had levelling-up initiatives for tackling digital poverty, which involved distributing IT hardware such as laptops to CYPF most in need or recycling old smartphones. CYPF who were refugees had, in some cases, been given a smartphone bought by their host families. At one site, HCPs reported that families changed their smartphone contract frequently to obtain the most competitive phone contract or deal, and this meant that, on occasions, their telephone number changed. Another difficulty occurred when families changed phone contracts, and the transfer of apps from the old to the new phone did not always happen. This meant CYPF had to download the DigiBete app to their new phone again and re-input the clinic code to access the app, which sometimes they did not do. This led to problems and an inability to carry on using the app.

An issue that emerged from our findings with the CYPF and HCPs was that, regardless of the family, while some preferred the app, the app did not replace face-to-face contact with HCPs. Rather, it was part of a “toolbox” of services offered by diabetes teams to help all CYPF manage T1DM. The literature indicates that CYPF frequently turn to trusted adults to help them make sense of online information and to provide alternative sources of support [24]. Therefore, face-to-face interactions, either virtual or digital, with HCPs remain important for all families when supporting them with their management of T1DM.

### 4.5. Limitations and Strengths

Learning from the process of conducting evaluations is important [27]. Limitations might indicate that the external validity of the results of this evaluation may be limited due to volunteer bias resulting from the non-probability sample and sample size. This limits the generalisability of the findings to other CYPF and HCPs, although similar determinants to the management of T1DM are likely to exist. We also recognize that the CYPF who engaged with the evaluation were more likely to be those using the app and were a self-motivated sample. Recruiting those not using the app, although difficult, would have provided a different perspective. This evaluation did not include an economic evaluation, which was not within the scope of this study but was conducted in a separate investigation [32]. Furthermore, this study did not assess the impact of the app on different insulin modalities or perform a comparative research design, so these are considerations for future investigations. People signed up to use the app on an individual basis, but we do not know if single accounts were accessed by multiple members of the same family. Not all app users participated in the evaluation. The app evaluation only covered five sites, so the generalisability of the findings might be limited. In addition, future investigations might assess cohorts in a prospective pattern comparing the knowledge, psychological condition, quality of life, and glycaemic control of the studied cohort before and after the use of the app.

The limitations of the evaluation are balanced by strengths and include the adoption of a mixed-methods evaluation design that collected quantitative and qualitative data at multiple NHS sites across England. The quantitative data were combined with qualitative data from four of the five sites to provide detailed insight into the impact and implementation of the DigiBete app. Furthermore, the evaluation is, to the best of our knowledge, the only service-level evaluation of an innovative app for helping CYPF and HCPs to improve the management of their T1DM. The accounts of CYPF and HCPs provided rich information on the impact and implementation of the app, including what works well and why and, importantly, what does not work as well and why, as is called for in the literature [14], this is important in improving interventions for CYPF with diabetes. The involvement of CYPF and HCPs in the evaluation is important in helping to improve the effectiveness and delivery of T1DM interventions [3]. With that in mind, information provided through the evaluation was shared with the developers of the DigiBete app in early 2023, so that refinements can be made to areas identified in this evaluation, that will help improve the care provided through the app. In doing so, this evaluation aspires to be beneficial and help improve the diabetes care of the CYPF who use the app. Moreover, our investigation provides insights into how we conducted this service-level evaluation that will be invaluable to other stakeholders contemplating and planning their own investigations.

## 5. Conclusions

T1DM is a major public health problem. However, structured interventions can help people living with the condition enhance their knowledge and skills and motivate them to take control and manage it effectively. In the understanding that face-to-face interventions are not ubiquitously effective, digital health interventions like DigiBete provide opportunities to optimise patient experience and outcomes while offering services in a more efficient way, reducing the burden on HCPs and proving cost effective.

Digital health interventions are proven to be effective in managing and preventing the occurrence of complications, improving self-efficacy [33], and facilitating improvements in HbA1c [34], especially when delivered through mobile apps or patient portals [35]. This evaluation found that the DigiBete app represents a trusted source of approved and regulated information, which provides a constant source of reassurance for CYPF. Moreover, HCPs validated DigiBete in helping CYPF to manage their T1DM while saving their service time and money. The evaluation was beneficial for informing learning [3,27] and also the delivery of the DigiBete app, that could subsequently shape future provision.

## Figures and Tables

**Table 1:** DigiBete app user information.

Site	Trust 1	Trust 2	Trust 3	Trust 4
App Users	188	246	294	411
App User Gender (* only male and female reported)	65% female, 34% male	50% female, 50% male	54% female, 46% male	54% female, 44% male
Average Diagnosis Length	4.0 years	3.1 years	3.7 years	4.2 years
Videos Viewed	776	1468	932	1679
Number of Quizzes Passed	207	136	293	266
Average Quiz Score	68%	69%	62%	61%
Awards Achieved	35	45	35	67

* Gender was collected and reported for male and female only and no other gender categories.

**Table 2:** Qualitative interview themes for CYPF and HCPs.

		CYPFs	HCPs
**Theme 1**	Acceptability of the app	√	√
**Theme 2**	Functionality of the app (How is it being used?)	√	√
**Theme 3**	App content	√	√
**Theme 4**	Behaviour change	√	√
**Theme 5**	DigiBete champions		√
**Theme 6**	Benefits of the app	√	√
**Theme 7**	Addressing inequalities	√	√
**Theme 8**	Potential app improvements	√	√
**Theme 9**	Resource savings associated with DigiBete		√

## Data Availability

The data presented in this study are available on request from the corresponding author. The data are not publicly available due to privacy or ethical restrictions.
